# Supplementary material for: Influence of the Antibiotic Oxytetracycline on the Morphometric Characteristics and Endophytic Bacterial Community of Lettuce (Lactuca sativa L.)
Source: Microorganisms. 2023 Nov 21;11(12):2828. doi: 10.3390/microorganisms11122828 (PMC10746115; doi:10.3390/microorganisms11122828)
Supplement: Supplementary file 1 [file microorganisms-11-02828-s001.zip › Supplement Table S1.pdf]

Table S1. The tables below display the results of difference significance analysis, calculated using the Mann-Whitney test ($p<0.05$) and the online calculator (https://www.socscistatistics.com/tests/mannwhitney/default2.aspx), where "+" – there are differences between samples, "–" –there are no differences between samples

**Figure 1a. Stems.**			
	control	OTC15	OTC50
control		+	+
OTC15	+		-
OTC50	+	-	
**Figure 1a. Roots.**			
	control	OTC15	OTC50
control		+	+
OTC15	+		-
OTC50	+	-	
**Figure 1b. Biomass**			
	control	OTC15	OTC50
control		+	+
OTC15	+		+
OTC50	+	+	
**Figure 1c. Chlorophyll content**			
	control	OTC15	OTC50
control		+	+
OTC15	+		+
OTC50	+	+	

**Figure 2.**			
	Leaf (control)	Root (control)	Water (control)
Leaf (control)		+	+
Root (control)	+		+
Water (control)	+	+	
	Leaf (OTC15)	Root (OTC15)	Water (OTC15)
Leaf (OTC15)		+	+
Root (OTC15)	+		-
Water (OTC15)	+	-	
	Leaf (OTC50)	Root (OTC50)	Water (OTC50)
Leaf (OTC50)		+	+
Root (OTC50)	+		+
Water (OTC50)	+	+	
**Figure 3a.**			
	Leaf (control)	Root (control)	Water (control)
Leaf (control)		+	+
Root (control)	+		+
Water (control)	+	+	
	Leaf (OTC15)	Root (OTC15)	Water (OTC15)
Leaf (OTC15)		+	+
Root (OTC15)	+		-
Water (OTC15)	+	-	
	Leaf (OTC50)	Root (OTC50)	Water (OTC50)
Leaf (OTC50)		+	+

Root (OTC50)	+		-
Water (OTC50)	+	-	

Figure 3b. Simpson indice

	Leaf (control)	Root (control)	Water (control)
Leaf (control)		-	+
Root (control)	-		+
Water (control)	+	+	

	Leaf (OTC15)	Root (OTC15)	Water (OTC15)
Leaf (OTC15)		-	+
Root (OTC15)	-		+
Water (OTC15)	+	+	

	Leaf (OTC50)	Root (OTC50)	Water (OTC50)
Leaf (OTC50)		+	+
Root (OTC50)	+		-
Water (OTC50)	+	-	

Shannon indice

	Leaf (control)	Root (control)	Water (control)
Leaf (control)		+	+
Root (control)	+		+
Water (control)	+	+	

	Leaf (OTC15)	Root (OTC15)	Water (OTC15)
Leaf (OTC15)		+	+
Root (OTC15)	+		+
Water (OTC15)	+	+	

	Leaf (OTC50)	Root (OTC50)	Water (OTC50)
Leaf (OTC50)		+	+
Root (OTC50)	+		-
Water (OTC50)	+	-	

Figure 4a.

	Leaf (OTC15)	Root (OTC15)
Leaf (OTC15)		+
Root (OTC15)	+	

	Leaf (OTC50)	Root (OTC50)
Leaf (OTC50)		+
Root (OTC50)	+	

Figure 4b.

	Root (OTC50)	Water (OTC50)
Root (OTC50)		+
Water (OTC50)	+	

Figure 5a. Stems

	control	OTC15	OTC300
control		-	+
OTC15	-		+
OTC300	+	+	

Figure 5a. Roots

	control	OTC15	OTC300

control		-	+
OTC15	-		+
OTC300	+	+	
Figure 5b. Biomass			
	control	OTC15	OTC300
control		-	+
OTC15	-		+
OTC300	+	+	
Figure 5c. Chlorophyll content			
	control	OTC15	OTC300
control		-	-
OTC15	-		-
OTC300	-	-	
**Figure 6.**			
	Leaf (control)	Root (control)	Soil (control)
Leaf (control)		+	+
Root (control)	+		+
Soil (control)	+	+	
	Leaf (OTC15)	Root (OTC15)	Soil (OTC15)
Leaf (OTC15)		+	+
Root (OTC15)	+		+
Soil (OTC15)	+	+	
	Leaf (OTC300)	Root (OTC300)	Soil (OTC300)
Leaf (OTC300)		+	+
Root (OTC300)	+		+
Soil (OTC300)	+	+	
**Figure 7a.**			
	Leaf (control)	Root (control)	Soil (control)
Leaf (control)		+	+
Root (control)	+		+
Soil (control)	+	+	
	Leaf (OTC15)	Root (OTC15)	Soil (OTC15)
Leaf (OTC15)		+	+
Root (OTC15)	+		+
Soil (OTC15)	+	+	
	Leaf (OTC300)	Root (OTC300)	Soil (OTC300)
Leaf (OTC300)		+	+
Root (OTC300)	+		+
Soil (OTC300)	+	+	
**Figure 7b. Simpson indice**			
	Leaf (control)	Root (control)	Soil (control)
Leaf (control)		-	-
Root (control)	-		-
Soil (control)	-	-	
	Leaf (OTC15)	Root (OTC15)	Soil (OTC15)
Leaf (OTC15)		-	-
Root (OTC15)	-		-
Soil (OTC15)	-	-	

	Leaf (OTC300)	Root (OTC300)	Soil (OTC300)
Leaf (OTC300)		-	-
Root (OTC300)	-		-
Soil (OTC300)	-	-	
**Shannon indice**			
	Leaf (control)	Root (control)	Soil (control)
Leaf (control)		-	+
Root (control)	-		+
Soil (control)	+	+	
	Leaf (OTC15)	Root (OTC15)	Soil (OTC15)
Leaf (OTC15)		-	+
Root (OTC15)	-		+
Soil (OTC15)	+	+	
	Leaf (OTC300)	Root (OTC300)	Soil (OTC300)
Leaf (OTC300)		+	+
Root (OTC300)	+		+
Soil (OTC300)	+	+	
**Figure 8a.**			
	Leaf (OTC15)	Root (OTC15)	Soil (OTC15)
Leaf (OTC15)		+	+
Root (OTC15)	+		+
Soil (OTC15)	+	+	
	Leaf (OTC300)	Root (OTC300)	Soil (OTC300)
Leaf (OTC300)		+	+
Root (OTC300)	+		+
Soil (OTC300)	+	+	
**Figure 8b.**			
	Root (OTC15)	Water (OTC15)	Soil (OTC15)
Root (OTC15)		+	+
Water (OTC15)	+		+
Soil (OTC15)	+	+	
	Leaf (OTC300)	Root (OTC300)	Soil (OTC300)
Leaf (OTC300)		+	+
Root (OTC300)	+		+
Soil (OTC300)	+	+	
