# Supplementary material for: Influence of the Antibiotic Oxytetracycline on the Morphometric Characteristics and Endophytic Bacterial Community of Lettuce (Lactuca sativa L.)
Source: Microorganisms. 2023 Nov 21;11(12):2828. doi: 10.3390/microorganisms11122828 (PMC10746115; doi:10.3390/microorganisms11122828)
Supplement: Supplementary file 1 [file microorganisms-11-02828-s001.zip › Supplement Table S2.pdf]

Table S2: High-throughput sequencing data analysis.

		sample name	sample-id	Raw reads	N of chimeras	Non-chimeric reads	Reads with OTU	OTU per sample	Faith's PD	chao1	observed_otus	shannon	simpson
experiment with hydroponics	water	W-Cont	1	45625	11697	33928	33193	1772	76.06831	2211.507614	1772	7.431143841	0.979094111
		W-Cont	2	46543	10023	30223	29995	1463	74.05586001	2511.567479	1357	7.877654877	0.965786959
		W-Cont	3	41655	9866	35677	30235	1855	70.03461078	2868.678875	1855	7.375826546	0.971235895
		W-OTC15	4	39360	6611	32749	32449	785	37.44875	1020.360656	785	5.068225593	0.921092377
		W-OTC15	5	32546	7514	28564	36897	725	35.12345	1256.455554	756	5.147855854	0.935465469
		W-OTC15	6	35254	7988	35549	28788	765	30.12389	1956.54787	705	5.014644457	0.924562124
		W-OTC50	7	33293	6567	26726	26527	476	19.24951	598.46	476	4.425201543	0.902556272
		W-OTC50	8	30542	7125	20056	20255	402	25.58454	369.57	386	4.945236779	0.929874563
		W-OTC50	9	35956	6233	19852	19665	436	21.47478	487.66	425	4.656664113	0.912578955
	leaf	LW-Cont	10	32971	192	32779	32752	242	16.40142	456.097561	242	1.619807291	0.484610258
		LW-Cont	11	38958	213	38745	38701	264	15.77534	578.2702703	264	1.631283769	0.49030914
		LW-Cont	12	29122	121	29001	28984	241	15.51292	483.3846154	241	1.70326453	0.505899909
		LW-OTC15	13	24544	108	24436	24421	170	9.52349	345.3846154	170	1.462524116	0.441184821
		LW-OTC15	14	20690	105	20585	20569	145	7.81525	238.8571429	145	1.555337096	0.471319522
		LW-OTC15	15	16465	134	23655	28257	205	10.34564	276.3647435	165	1.315556786	0.406874651
		LW-OTC50	16	15263	55	15208	15193	156	15.90369	346.0434783	156	1.433566568	0.411877612
		LW-OTC50	17	18642	96	13254	19423	184	12.35684	396.564858	204	1.256474585	0.556432165
		LW-OTC50	18	12656	102	17514	16544	172	14.53486	421.3546847	186	1.554684651	0.498946545
	root	RW-Cont	19	12947	48	12899	12881	175	15.66044	373	175	1.720692595	0.498381652
		RW-Cont	20	32645	181	32464	32375	348	29.99006	467.4358974	348	1.965670426	0.519864687
		RW-Cont	21	36938	148	36790	36744	394	24.38093	561.037037	394	2.161723393	0.549737638
		RW-OTC15	22	22680	86	22594	22560	260	18.06005	401.375	260	1.88626301	0.521228642
		RW-OTC15	23	32489	281	32208	32146	331	21.86129	458.3943662	331	1.963830635	0.565195256
		RW-OTC15	24	30545	181	28546	29545	301	20.54555	413.6546465	303	1.901884567	0.54545469
		RW-OTC50	25	23139	778	22361	22203	367	25.04266	439.6785714	367	3.759702032	0.770605784
		RW-OTC50	26	19563	712	29554	36441	287	23.56121	499.6543217	307	3.554546848	0.745465455
		RW-OTC50	27	29554	806	19664	26222	308	20.35453	387.6546515	430	3.704654168	0.786547888

experiment with soil	root	RS-Cont	28	114185	126	114059	114011	294	19.85685	394.2580645	294	1.042030745	0.278402019
		RS-Cont	29	132036	181	131855	131812	372	25.06248	476.2051282	372	0.988836717	0.256811959
		RS-Cont	30	64523	104	64419	64385	277	21.47614	436.6	277	1.046589616	0.287839555
		RS-OTC15	31	65150	49	65101	65075	239	20.32363	346.4468085	239	0.85127585	0.208411117
		RS-OTC15	32	74858	52	74806	74755	309	22.7524	492.9574468	309	0.949796646	0.224502065
		RS-OTC15	33	47207	26	47181	47160	190	17.30089	300.6756757	190	0.990996661	0.258410488
		RS-OTC300	34	46473	36	46437	46413	256	22.50009	372.4347826	256	0.99246563	0.237250725
		RS-OTC300	35	47835	69	47766	47735	280	23.61061	507.2195122	280	1.015604635	0.244444379
		RS-OTC300	36	59607	23	59584	59557	254	20.55766	386.0769231	254	0.907163522	0.202961771
	leaf	LS-Cont	37	48158	49	48109	48082	132	12.92839	267.05	132	0.510500584	0.12379832
		LS-Cont	38	53287	62	53225	53199	127	12.26791	186.625	127	0.531614339	0.130947911
		LS-Cont	39	51391	36	51355	51338	135	13.71215	198.3703704	135	0.46801724	0.105289629
		LS-OTC15	40	42337	71	42266	42249	116	11.55945	193	116	0.47954937	0.109240331
		LS-OTC15	41	52376	43	52333	52311	150	12.80193	316.05	150	0.528141064	0.120660016
		LS-OTC15	42	60690	28	60662	60639	143	14.13383	206.030303	143	0.396533021	0.080472062
		LS-OTC300	43	64558	43	64515	64496	139	12.67732	244.12	139	0.375819512	0.079906713
		LS-OTC300	44	68334	34	68300	68277	139	12.51579	289.3529412	139	0.304883874	0.057637386
		LS-OTC300	45	61744	41	61703	61682	128	10.24531	189.03125	128	0.338697226	0.071305934
	soil	S-OTC15	46	53741	894	52847	52479	5701	199.23945	7304.302045	5701	9.967879195	0.987174276
		S-OTC15	47	71497	387	71110	70789	5494	196.129	6423.334416	5494	9.704116497	0.983644407
		S-OTC15	48	60525	319	60206	59902	5189	188.03664	6156.667079	5189	10.35059714	0.996531649
		S-OTC300	49	85836	2903	82933	82419	6179	207.1977	7808.921569	6179	9.700119935	0.990157073
		S-OTC300	50	67038	462	66576	66195	5577	196.38462	6705.826471	5577	9.782991238	0.98623358
		S-OTC300	51	74396	330	74066	73763	4855	172.29061	5634.505988	4855	9.881852771	0.990073431
		S-Cont	52	41777	4274	37503	36153	5119	179.28133	6101.670103	5119	10.35684757	0.993458879
		S-Cont	53	53564	4170	49394	48303	5003	177.84563	6015.962857	5003	9.70191974	0.988991102
		S-Cont	54	64071	4381	59690	58453	5856	199.82035	6790.266282	5856	10.1253905	0.988683704
